# Construct validity of the atopic dermatitis control tool in the pediatric population: A prospective study

**DOI:** 10.1016/j.jdin.2025.08.010

**Published:** 2025-09-26

**Authors:** Rivka Friedland, Athar Beshara, Yael Anne Leshem, Yael Renert-Yuval

**Affiliations:** a Pediatric Dermatology Unit, Schneider Children's Medical Center of Israel, Petah Tikva, Israel; b Gray Faculty of Medical and Health Sciences, Tel Aviv University, Tel Aviv, Israel; c Division of Dermatology, Rabin Medical Center, Beilinson Hospital, Petah Tikva, Israel

**Keywords:** atopic dermatitis, atopic dermatitis control tool, patient-reported outcomes, pediatric patients

*To the Editor:* Patient-reported outcomes (PROs) are key for atopic dermatitis (AD) management, including the AD control tool (ADCT).^1^^,^^2^ Although AD primarily affects pediatric patients,^3^ ADCT validation in children is lacking.^1^

To determine the construct validity of the ADCT in children,^4^ we conducted a real-world study of pediatric AD patients comparing parent- and child-reported (where applicable) ADCT scores (P-ADCT and C-ADCT, respectively) with other PROs: pruritus and insomnia numerical rating score, and with clinician-reported outcomes (ClinRO): body surface area, investigator’s global assessment, Eczema Area and Severity Index, and investigator’s global assessment∗body surface area. Patients were assessed and enrolled in person during a pediatric dermatology clinic visit after providing an institutional review board informed consent. The ADCT was administered using official language translations, based on parent and patient preferences. Spearman correlations were performed with a priori hypotheses for construct validity defined as r ≥0.5 for PRO and r = 0.30-0.50 for ClinRO assessments. Statistical analysis was performed with R software (www.R-project.org).

P-ADCT, PROs, and ClinRO were completed for 100 children (mean age 7.0 ± 4.7 years) (Table I). Twenty-five patients (mean age 13.2 ± 2.3 years) also self-reported C-ADCT (and thus had paired P-ADCT, in contrast to those with unpaired P-ADCT, who were mostly younger patients, unable to self-report ADCT). Since parents completed PRO only when the child could not self-report, parent-PRO data were unavailable for self-reporting children.

**Table I:** Patient characteristics

	Patients with only P-ADCT (*N* = 75)	Children self-reporting ADCT (*N* = 25)	All (*N* = 100)
Age, y (SD)	5.0 (3.4)	13.2 (2.3)	7.0 (4.7)
Gender, M (%)	40 (53%)	10 (38%)	50 (50%)
IGA, mean (SD)	2.7 (1.0)	2.4 (1.0)	2.7 (1.0)
EASI, mean (SD)	14.4 (12.9)	14.3 (16.1)	14.4 (13.7)
BSA, mean (SD)	25.8 (21.6)	21.6 (26.8)	24.7 (24.8)
Total P-ADCT, mean (SD)	14.9 (6.7)	11.9 (6.6)	14.2 (6.8)
Total C-ADCT, mean (SD)	NA	9.1 (5.5)	NA
Insomnia NRS, mean (SD)	5.6 (3.4)∗	3.9 (3.6)†	5.1 (3.5)
Pruritus NRS, mean (SD)	6.9 (2.6)∗	5.6 (2.5)†	6.6 (3.5)

*ADCT*, Atopic dermatitis control tool; *BSA*, body surface area; *C-ADCT*, child-reported atopic dermatitis control tool; *EASI*, Eczema Area and Severity Index; *IGA*, investigator global assessment; *NA*, not applicable; *NRS*, numerical rating score; *P-ADCT*, parent-reported atopic dermatitis control tool.

∗ Only parent-reported.

† Only child-reported.

ADCT dimensionality was evaluated using exploratory factor analysis, which consistently supported a unidimensional structure across P-ADCT (full cohort, paired, and unpaired) and C-ADCT subgroups (Supplementary Tables I and II, Fig 1, available via Mendeley at https://data.mendeley.com/preview/wc6mgbf58r?a=53a46bd5-b15e-40d5-a737-edb9933cd4be). Internal consistency was high for the P-ADCT (Cronbach’s α = 0.903, 95% CI: 0.870-0.936; standardized α = 0.912), with similar reliability in unpaired (α = 0.903) and paired (α = 0.905) subgroups. The C-ADCT also showed strong reliability (α = 0.867, 95% CI: 0.787-0.947; standardized α = 0.873).

Spearman correlations for both total scores and individual P-ADCT/C-ADCT items are presented in Table II, with additional known-groups analysis shown in Supplementary Fig 2, available via Mendeley at https://data.mendeley.com/preview/wc6mgbf58r?a=53a46bd5-b15e-40d5-a737-edb9933cd4be. Overall, correlations with total P-ADCT/C-ADCT scores met our criteria for construct validity: stronger correlations were recorded with other PROs (pruritus/insomnia, r = 0.64-0.79), as compared with ClinRO (r = 0.44-0.64). Total P-ADCT displayed adequate-to-good correlation with total C-ADCT (r = 0.70) (Supplementary Table III, available via Mendeley at https://data.mendeley.com/preview/wc6mgbf58r?a=53a46bd5-b15e-40d5-a737-edb9933cd4be).

**Table II:** Spearman correlations across total and individual ADCT items and PRO/ClinRO∗

	Patients with only P-ADCT (*N* = 75)	Children self-reported C-ADCT (*N* = 25)	P-ADCT of patients with C-ADCT (*N* = 25)
ClinRO corr. with total ADCT (mean)	0.44-0.59 (0.50)	0.44-0.64 (0.56)	0.49-0.67 (0.58)
ClinRO corr. with ADCT I (mean)	0.32-0.52 (0.42)	0.51-0.70 (0.61)	0.56-0.64 (0.59)
ClinRO corr. with ADCT II (mean)	0.36-0.52 (0.43)	0.54-0.72 (0.63)	0.41-0.68 (0.56)
ClinRO corr. with ADCT III (mean)	0.43-0.59 (0.51)	0.26-0.49 (0.39)	0.24-0.46 (0.37)
ClinRO corr. with ADCT IV (mean)	0.45-0.49 (0.47)	0.68-0.76 (0.72)	0.50-0.64 (0.58)
ClinRO corr. with ADCT V (mean)	0.35-0.49 (0.40)	0.19-0.28 (0.22)	0.46-0.64 (0.56)
ClinRO corr. with ADCT VI (mean)	0.38-0.51 (0.44)	0.06-0.20 (0.14)	0.37-0.47 (0.43)
PRO corr. with total ADCT (mean)	0.72-0.78 (0.75)	0.66-0.71 (0.68)	NA
PRO corr. with ADCT I (mean)	0.51-0.52 (0.52)	0.66-0.73 (0.69)	
PRO corr. with ADCT II (mean)	0.50-0.56 (0.53)	0.67-0.68 (0.67)	
PRO corr. with ADCT III (mean)	0.72-0.73 (0.72)	0.55-0.56 (0.55)	
PRO corr. with ADCT IV (mean)	0.62-0.80 (0.71)	0.67-0.80 (0.73)	
PRO corr. with ADCT V (mean)	0.70-0.71 (0.71)	0.18-0.33 (0.25)	
PRO corr. with ADCT VI (mean)	0.50-0.60 (0.55)	0.31-0.50 (0.41)	

ClinRO includes EASI, IGA, BSA, and IGA∗BSA. PRO includes NRS for insomnia and pruritus.

*ADCT*, Atopic dermatitis control tool; *BSA*, body surface area; *C-ADCT*, child-reported ADCT; *ClinRO*, clinician-reported outcomes; *EASI*, Eczema Area and Severity Index; *IGA*, investigator global assessment; *NA*, not applicable; *NRS*, numerical rating score; *P-ADCT*, parent-reported ADCT; *PRO*, patient-reported outcome.

∗ A priori hypotheses for construct validity defined as r ≥0.5 for PRO and r = 0.30-0.50 for ClinRO. PROs were completed by the parent when the child could not self-report and by the child when a C-ADCT was completed. NA indicates that parent-reported PROs were not available for children who self-reported.

Across all P-ADCT/C-ADCT individual items, C-ADCT item IV (difficulty sleeping) displayed the highest correlations with ClinRO (r = 0.68-0.76). In contrast, 2 C-ADCT items, item V (effect on daily activities) and item VI (effect on mood/emotions), displayed below-threshold correlations with both ClinRO and child-PRO: r = 0.19-0.29 and r = 0.06-0.20 for ClinRO, and r = 0.18-0.33 and r = 0.31-0.50 for child-PRO, respectively (Table II).

This study's generalizability may be limited by the predominance of moderate-to-severe patients in a tertiary pediatric dermatology clinic setting. Additionally, the C-ADCT subgroup was relatively small. Nonetheless, it provides new evidence relevant to the utility of the ADCT in pediatric patients, including a multifaceted evaluation of structure and item performance.

In summary, our results support the use of the ADCT in pediatric AD, demonstrating adequate construct validity when reported by both parents and children. While parents’ ADCT performed well across all items, conceptual items (daily activity/mood/emotions) displayed poor correlations when children completed the ADCT. Our findings underscore the importance of tailoring PRO tools to pediatric patients to optimize their utility in this population^5^ and support further investigation of the ADCT’s reliability over time and responsiveness in this age group.

## Conflicts of interest

None disclosed.
